# Chikungunya: epidemiology

**DOI:** 10.12688/f1000research.7171.1

**Published:** 2016-01-19

**Authors:** Lyle R. Petersen, Ann M. Powers

**Affiliations:** 1 Division of Vector-Borne Diseases, Centers for Disease Control and Prevention, Fort Collins, CO, USA

**Keywords:** Chikungunya, mosquito, alphavirus

## Abstract

Chikungunya virus is a mosquito-borne alphavirus that causes fever and debilitating joint pains in humans. Joint pains may last months or years. It is vectored primarily by the tropical and sub-tropical mosquito, *Aedes aegypti*, but is also found to be transmitted by *Aedes albopictus*, a mosquito species that can also be found in more temperate climates. In recent years, the virus has risen from relative obscurity to become a global public health menace affecting millions of persons throughout the tropical and sub-tropical world and, as such, has also become a frequent cause of travel-associated febrile illness. In this review, we discuss our current understanding of the biological and sociological underpinnings of its emergence and its future global outlook.

## Introduction

As with several other mosquito-borne alphaviruses, chikungunya virus causes a fever-rash-arthralgia syndrome in humans. The name chikungunya derives from the debilitating joint pain noted by local populations during an outbreak in 1952–53 in what is now Tanzania. The local word “chikungunya”, meaning “that which bends up” was given as a result of the stooped posture that resulted from the pain of the disease ^1, 2^. The arthralgia can persist for months or even years in some affected persons and can progress to frank arthritis in some ^3– 15^. Symptoms typically occur in 72–97% of those infected ^15– 19^, but one study, in an area where the virus has been long established endemically, showed that only 18% of infections resulted in clinical illness, possibly due to re-exposure events that did not result in clinical illness ^20^. Since 2004, massive urban outbreaks producing considerable morbidity in a widening geographical area have occurred throughout the topical and sub-tropical world. In this review, we will explore the complex interplay of entomological, virological, and sociological factors contributing to its emergence, speculate on future epidemiological trends, and outline the possibilities for control.

## Transmission cycles and mosquito vectors

Three chikungunya viral genotypes are recognized, which historically have circulated in the distinct geographical regions for which they are named: West African genotype, East Central South African (ECSA) genotype, and Asian genotype ^21^. Phylogenetic evidence suggests that the Asian genotype virus derived from the ECSA virus sometime between 1879 and 1927 ^22^. In Africa, the virus is maintained in a sylvatic cycle involving non-human primates and forest-dwelling *Aedes* spp. mosquitoes ^23^. In these rural regions, human outbreaks tend to be small and dependent on environmental conditions (such as increased rainfall) that increase sylvatic mosquito densities, particularly of the *Aedes furcifer-tayleri* group ^23, 24^. A sylvatic transmission cycle has not been identified in Asia, but is likely present due to ongoing, low level, human activity.

Like the arthropod-borne viruses (arboviruses) dengue, yellow fever, o’nyong’nyong, and Zika, humans are not dead-end hosts for chikungunya virus but rather serve as part of the transmission cycle by efficiently infecting *Aedes aegypti* mosquitoes. This property enables rapid human-mosquito-human transmission cycles in urban areas, which can produce massive outbreaks. *Aedes aegypti* is a highly efficient urban vector because it preferentially bites humans and often bites multiple humans in the course of acquiring a complete, single blood meal. Furthermore, *Aedes aegypti* breeds in the ubiquitous small pools of water found around human habitation, often bringing the vectors in close proximity to human hosts facilitating further transmission ^25, 26^.

In addition to *Aedes aegypti*, other mosquito vectors must be considered. Of particular interest is *Aedes albopictus* (Asian tiger mosquito), an aggressive, human-biting mosquito that has spread globally from its native Asia largely from international trade in used tires and other commodities in recent years ^27, 28^. Unlike *Aedes aegypti*, which exists in tropical and subtropical areas, *Aedes albopictus* can also thrive in temperate regions, thus potentially introducing chikungunya virus to new ecological niches ^29, 30^. Furthermore, other members of the *Stegomyia* subgenus may be important under certain circumstances. *Aedes henselli* was the principal vector of a large outbreak on Yap Island in Micronesia ^31, 32^. Other species present in focal areas may be of local importance.

## Early outbreaks

Although chikungunya virus cannot be definitively linked to outbreaks before its discovery in the mid-1950s, outbreaks of fever and debilitating polyarthralgia affecting a substantial proportion of the affected population were noted in many areas during the 18 ^th^ and 19 ^th^ centuries, including Africa, Asia, India, the West Indies, Indonesia, and the southern United States ^33, 34^. Interestingly, contemporary physicians called this disease “dengue” and noted its clinical similarities as well as its differences between what is currently known as “dengue” and “breakbone fever”. In the mid-1950s, several authors (including Sabin) ^35^ isolated the “breakbone fever” viruses and called them “dengue viruses”, and the former “dengue” became “chikungunya” following the 1952–53 outbreak that produced the first isolation of chikungunya virus. While some of these previous historical outbreaks could have been due to chikungunya virus, they also could have been caused by other viruses already present in specific locations and causing the same clinical syndrome, such as Mayaro virus in the Americas or o’nyong’nyong virus in Africa.

Following the discovery of chikungunya virus, numerous small outbreaks were noted in Africa. However, massive outbreaks were noted in Thailand in the late 1950s and early 1960s ^36, 37^, and in India from the early 1960s into the 1970s ^38^. Approximately 31% of the population of Bangkok became infected during the 1962 outbreak ^36^. Antibody prevalence ranged from 10–20% of the 1–2 year olds to 70–85% among adults, suggesting long-standing endemicity in that area. High attack rates were noted during outbreaks in Madras (40%) in 1962–64 and in Barsi (37%) in 1973 ^39^. For unknown reasons, outbreaks in India abruptly stopped, not to reoccur for the next 32 years ^40^.

## Current epidemic

### Causative factors

The current epidemic, ongoing since 2004, involves many tropical and sub-tropical areas of Africa, Asia, Europe, the Pacific archipelago, and the Americas. Both ECSA and Asian genotype viruses, sometimes together, are responsible for epidemics, depending on location. The fact that both genotypes have nearly simultaneously re-emerged after years of relatively little activity suggests that similar forces may be driving their re-emergence. This hypothesis is reinforced by our recent experience of substantial geographical spread and massive increased disease incidence of the four dengue serotype viruses which, as noted earlier, are vectored also by *Aedes aegypti* mosquitoes in human-mosquito-human urban transmission cycles ^41^. Factors attributed to dengue emergence include increased human travel, urbanization of human populations, uncontrolled urban growth (leading to increases in *Aedes aegypti* breeding sites), and lack of adequate control measures ^42, 43^. While dengue incidence in the Americas has been increasing for more than three decades, large increases have occurred in the last decade, suggesting an epidemiological turning point highly permissive for the transmission and spread of *Aedes aegypti*-vectored arboviruses. This point was recently reinforced by the spread throughout the Pacific ^44, 45^ and into the Americas (Brazil) ^46, 47^ for the first time of Zika virus, a flavivirus also spread from human to human *via Aedes aegypti*.

### Re-emergence of the ECSA genotype

As mentioned previously, chikungunya outbreaks now occurring globally are caused by both ECSA and Asian genotypes. The current ECSA outbreak began on Lamu Island on coastal Kenya in 2004 ^11^. This outbreak involved an estimated 13,500 persons, which was quite substantial compared to other contemporary African chikungunya outbreaks. Eight months later on Comoros, an island off the coast of Tanzania, an outbreak involved nearly 215,000 of that island’s residents ^48^. Phylogenetic analysis showed that the causative ECSA genotype virus was nearly genetically identical to the Lamu Island outbreak, suggesting that the Comoros outbreak was simply an extension of the Lamu Island outbreak ^49^. A small outbreak on La Reunion Island also began in 2005, an island with very low *Aedes aegypti* populations. Atypical of recent chikungunya outbreaks, this outbreak smouldered until December 2005 when incidence dramatically increased, eventually involving >244,000 persons ^50^. Viral isolates collected in 2006 contained an envelope protein mutation (E1: A226V) that was found to increase viral fitness in *Aedes albopictus* mosquitoes ^51, 52^, which led to the hypothesis that the lack of abundant *Aedes aegypti* populations produced the outbreak’s slow onset, but incidence picked up when the viral mutation increased transmission efficiency for the abundant *Aedes albopictus* mosquito.

The ECSA virus containing the E1: A226V mutation subsequently spread from La Reunion Island to India by 2006, where over 1 million cases were reported in the first year alone ^53^. Activity in India still continues nearly a decade later. From India, the Indian Ocean lineage strain spread to Southeast Asia and to northern Italy ^17, 54^. The Italian outbreak, which involved approximately 300 persons, was significant as this was the first outbreak documented in a subtropical climate in an area where the only vector species was *Aedes albopictus*.

Autochthonous transmission of an ECSA genotype virus was identified for the first time in the Americas in Brazil in 2014 ^55^. Subsequently, this virus has spread to multiple areas in that country. The index case was a traveller from Angola and the virus does not contain the E1: A226V mutation, suggesting that it might have limited infectivity for *Aedes albopictus*.

### Geographical expansion of the Asian genotype

The Asian genotype has spread throughout the Pacific in recent years. A small 2011 outbreak in New Caledonia began after two infected travellers returned from Indonesia where the virus had been circulating continuously for at least a decade ^56^. The Asian genotype subsequently was noted during outbreaks in the following locations: Papua New Guinea (2012); Yap Island, Federated States of Micronesia (2013); Tonga, Samoa, American Samoa, Tokelau, French Polynesia (2014); and Kiribati and the Cook Islands (2015) ^44, 47, 57^.

**Figure 1.**
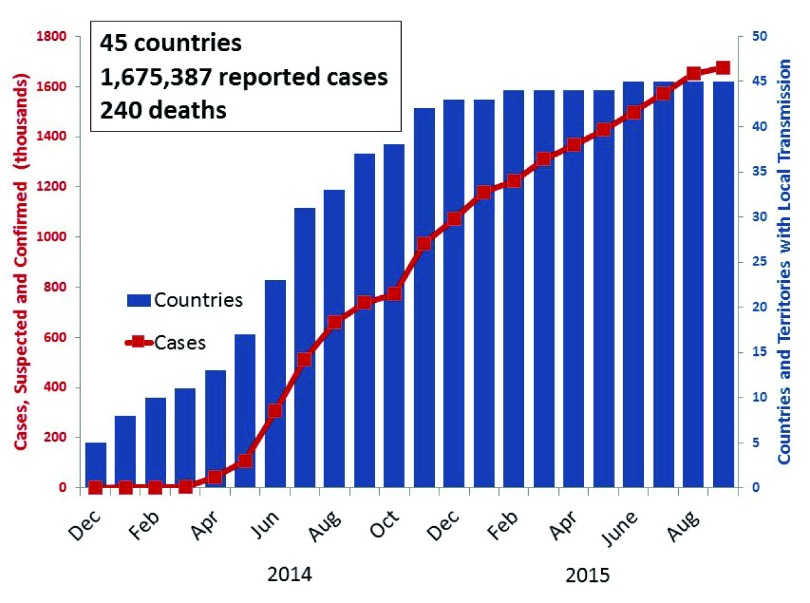
Number of countries in the Americas with local chikungunya transmission and number of cases reported to the Pan American Health Organization, by month, December 2013–September 2015.

However, the most dramatic expansion occurred in the Americas where autochthonous transmission began on the Island of St. Martin in 2013 ^58^. Genetic sequencing indicated that the virus was similar to chikungunya viruses recently identified from the Federated States of Micronesia, Philippines, and Indonesia ^59^. Within a year, the virus spread to 26 islands and 14 mainland countries, resulting in more than 1 million reported cases. As of September 2015, 1.7 million cases and 240 deaths were reported from 45 of the 53 countries or territories reporting to the Pan American Health Organization ( Figure 1). The true number of affected persons is undoubtedly substantially higher; since laboratory confirmation is completed in only a fraction of cases, infected individuals often do not seek medical care, and reporting may be incomplete. For example, Cuba has not reported any cases, yet infected travellers have returned to the United States from that country. While activity in many island countries in the Caribbean has decreased or stopped, transmission continues in most mainland countries.

In 2014, a total of 2788 cases were reported among travellers returning to the United States, nearly all from the Caribbean and Latin America. Despite this large influx, only 11 instances of autochthonous transmission have been identified in the contiguous United States, all in Florida ^60^.

## Future outlook

### Tropical areas

The continuing anthropogenic factors promoting the emergence and spread of chikungunya and other *Aedes aegypti*-borne diseases suggest that the current global epidemic will continue to spread to previously unaffected areas for some time. However, as the outbreak continues, herd immunity in humans will eventually curtail the scope and frequency of these outbreaks since humans are the virus’s only significant vertebrate host in urban settings and life-long immunity follows infection. The level of herd immunity required to stop outbreaks is unknown and likely varies according to local underlying transmission dynamics, such as human population size and mosquito abundance. Studies demonstrated that upwards of 60% of the population became infected during some outbreaks ^48^, after which outbreaks ceased or were greatly reduced. One or two transmission seasons seem to be sufficient to curtail transmission activity in small, isolated populations such as islands, as has occurred in smaller Pacific Islands recently and now appears to be occurring in some Caribbean islands. At the other extreme, outbreaks in India, with its large and dispersed population, have moved from place to place now for nearly a decade, as they had in the early 1960s and 1970s. However, even in India, the reduction in susceptible populations had been apparently sufficient to prevent another large outbreak for 32 years.

In the Americas, several critical questions remain. As mentioned previously, the eventual duration and extent of epidemic activity is unknown, but will likely vary geographically. It is also unknown whether a sylvatic cycle will develop, enabling viral persistence without human-to-human transmission. The current outbreak’s substantial geographical reach and incidence make enduring sylvatic transmission a likely possibility if a mechanism exists for it to do so. Another question is to what extent the ECSA genotype introduced in Brazil will spread, which has important implications discussed later.

### Subtropical and temperate areas

The potential for large **chikungunya virus** outbreaks on the fringes of the *Aedes aegypti* distribution, such as the southern United States, seems limited given our experiences with dengue ^61^. Hundreds of dengue-infected travellers entering the contiguous United States are reported each year ^62^, but *Aedes aegypti*-vectored dengue outbreaks have been relatively infrequent, focal and self-limited. One exception was a dengue outbreak spanning two years and involving several hundred persons in Key West, Florida, the most southern point in the contiguous United States ^63^. However, most dengue outbreaks have occurred in Texas when large outbreaks in northern Mexico spilled over into towns along the border. Investigations in Texas showed that sociological conditions that limit contact with the indoor-biting *Aedes aegypti* mosquito, such as the use of air conditioning, greatly limited transmission despite the presence of abundant *Aedes aegypti* populations ^63– 66^. Dengue outbreaks have not occurred in areas with *Aedes albopictus* but without *Aedes aegypti* in the contiguous United States.

So far, it appears that chikungunya virus is following a similar pattern to dengue in subtropical and temperate areas of the United States. The thousands of infected returning travellers have produced only a handful of identified autochthonous transmission cases and no outbreaks. This dynamic could change, however, if large chikungunya outbreaks in northern Mexico spill over into border towns, as has occurred for dengue.

Of some concern, *Aedes albopictus* is endemic in much of the southern and eastern United States ^67^ and laboratory experiments show that *Aedes albopictus* mosquitoes collected throughout the Americas are generally competent to transmit the Asian genotype chikungunya virus ^68, 69^. Nevertheless, to date the Italian outbreak of 2007 is the only documented subtropical outbreak vectored solely by *Aedes albopictus* and this involved the ECSA virus with the E1: A226V mutation ^54^. The ECSA genotype now circulating in Brazil may be less competent for transmission by *Aedes albopictus* mosquitoes than ECSA strains circulating in the Old World, as it does not possess the E1: A226V mutation, but it could prove problematic if the virus acquires mutations that increase vector competence and is introduced to subtropical and temperate areas endemic for *Aedes albopictus*.

## Options for control

Historically, control of mosquito-transmitted viruses has relied heavily upon efforts aimed at reducing mosquito populations. These control activities focus on eliminating mosquito larval habitat and adulticiding. However, because *Aedes aegypti* and *Aedes albopictus* mosquitoes are container-breeding species that will lay eggs in nearly any water habitat, larval control efforts are a challenging, if not impossible, task ^70^. Control efforts aimed at reducing adult mosquito populations also fail, because truck-based or aerial pesticide applications do not reach many adults, which rest and bite indoors. Indoor residual pesticide spraying might be effective, but is impractical in large urban areas. In addition, the threshold *Aedes aegypti* population levels required to stop chikungunya transmission are unknown, but are likely quite low. Ominously, Singapore, a country with perhaps the most effective *Aedes aegypti* control program globally, has experienced large chikungunya outbreaks vectored by both *Aedes aegypti* and *Aedes albopictus* ^71, 72^. Several new *Aedes aegypti* control approaches are under development, but none are currently ready for widespread use ^73– 75^.

An alternative control option is the development of a chikungunya vaccine. Several platform options have been explored ranging from virus-like particles, live attenuated variants, virus-vectored products, subunit vaccines, DNA vaccines, or inactivated products ^76^. At least nine distinct options have been examined in pre-clinical research with most demonstrating the potential for protection in animal model systems. Only three have been tested in clinical trials: a live attenuated product developed by scientists at the Walter Reed Army Institute of Research (WRAIR) in the 1980s and 1990s ^77, 78^, a virus-like particles product developed recently at the National Institutes of Health ^79^, and a measles-vectored product expressing the **chikungunya virus** structural genes ^80^. While all appear to be promising candidates, the likelihood of a product reaching a commercial market are slim, due to the unpredictable nature of **chikungunya virus** outbreaks, the challenges associated with performing efficacy trials, and the uncertainty of revenue generating capacity. However, despite the challenges, a vaccine is one of the best options for preventing further outbreaks.

## Concluding remarks

Urbanization, human travel, viral adaption, lack of effective control measures, and spread of new vectors likely have contributed to recent re-emergence of chikungunya. The current global outbreak, unprecedented in its size and geographical scope, is comprised of many smaller outbreaks that extend from place to place *via* human movement, and continue unabated until sufficient herd immunity in local human populations develops, or changes in other conditions, such as weather, inhibit further transmission. In smaller, island populations only one or two transmission seasons seem sufficient to greatly curtail or eliminate transmission; whereas, in larger populations such as India, transmission may extend beyond a decade. Like many other mosquito-borne diseases, it is very difficult to predict if and when a chikungunya outbreak will occur in any given location. Another uncertainty is the risk of epidemics in subtropical and temperate regions of the world where *Aedes albopictus* is a potential vector. The Italian outbreak demonstrated the possibility of such outbreaks, but to date outbreaks have yet to materialize in the United States, despite thousands of imported cases. While new chikungunya vaccines seem an attractive control possibility, many obstacles exist for their eventual commercialization. The dramatic spread of the dengue, chikungunya, and Zika viruses in recent years highlights the urgent need to identify scalable and cost-effective *Aedes aegypti* control options.

## Disclaimer

The findings and conclusions in this report are those of the author(s) and do not necessarily represent the official position of the Centers for Disease Control and Prevention.
